# [2,6-Bis(4,5-dihydro-1*H*-imidazol-2-yl)pyridine]dichloridomanganese(II)

**DOI:** 10.1107/S1600536809014354

**Published:** 2009-04-25

**Authors:** Chun-Xia Ren, Su-Yun Li, Zhao-Zhong Yin, Xiang Lu, Yu-Qiang Ding

**Affiliations:** a School of Chemical and Materials Engineering, Jiangnan University, 1800 Lihu Road, Wuxi, Jiangsu Province 214122, People’s Republic of China; b Department of Public Education, Jiangxi Vocational and Technical College of Electricity, 8 Mailu Road, Nanchang, Jiangxi Province 330032, People’s Republic of China

## Abstract

In the title compound, [MnCl_2_(C_11_H_13_N_5_)], the Mn^II^ ion is five-coordinated in a distorted square-pyramidal geometry, with three N atoms from the neutral tridentate 2,6-bis­(4,5-dihydro-1*H*-imidazol-2-yl)pyridine ligand and one chloride ion forming the basal plane and the other chloride ion in the apical position. Both dihydro­imidazole rings adopt envelope conformations. In the crystal structure, mol­ecules are linked into a three-dimensional network by N—H⋯Cl and C—H⋯Cl hydrogen bonds.

## Related literature

For the synthesis of 2,6-bis­(4,5-dihydro-1*H*-imidazol-2-yl)­pyridine, see: Baker *et al.* (1991 ▶). For general background, see: Bordo *et al.* (2001 ▶); Hagrman *et al.* (1999 ▶); Yaghi *et al.* (1998 ▶). For related structures, see: Böca *et al.* (2005 ▶); Haga *et al.* (1996 ▶); Hammes *et al.* (2005 ▶); Ren, Ye, He *et al.* (2004 ▶); Ren, Ye, Zhu *et al.* (2004 ▶); Ren *et al.* (2007 ▶); Stupka *et al.* (2004 ▶); Sun *et al.* (2008 ▶).
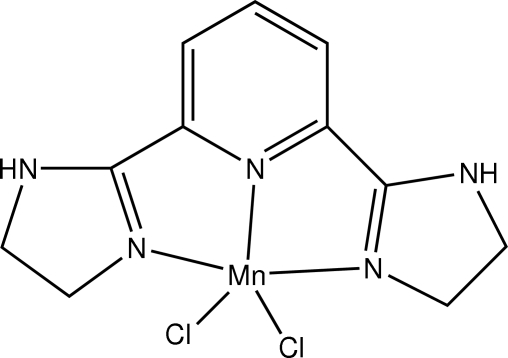


## Experimental

### 

#### Crystal data

[MnCl_2_(C_11_H_13_N_5_)]
*M* *_r_* = 341.10
Monoclinic, 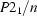

*a* = 9.297 (5) Å
*b* = 12.686 (7) Å
*c* = 12.383 (6) Å
β = 100.313 (9)°
*V* = 1436.9 (13) Å^3^
*Z* = 4
Mo *K*α radiation
μ = 1.28 mm^−1^
*T* = 273 K
0.30 × 0.25 × 0.21 mm

#### Data collection

Bruker SMART CCD area-detector diffractometer
Absorption correction: multi-scan (*SADABS*, Bruker, 1998 ▶) *T*_min_ = 0.700, *T*_max_ = 0.774
8507 measured reflections
3317 independent reflections
1750 reflections with *I* > 2σ(*I*)
*R*_int_ = 0.077

#### Refinement

*R*[*F* ^2^ > 2σ(*F* ^2^)] = 0.067
*wR*(*F* ^2^) = 0.187
*S* = 0.95
3317 reflections
173 parameters
H-atom parameters constrained
Δρ_max_ = 0.68 e Å^−3^
Δρ_min_ = −0.53 e Å^−3^

### 

Data collection: *SMART* (Bruker, 1998 ▶); cell refinement: *SAINT-Plus* (Bruker, 1998 ▶); data reduction: *SAINT-Plus*; program(s) used to solve structure: *SHELXS97* (Sheldrick, 2008 ▶); program(s) used to refine structure: *SHELXL97* (Sheldrick, 2008 ▶); molecular graphics: *SHELXTL* (Sheldrick, 2008 ▶); software used to prepare material for publication: *SHELXL97*.

## Supplementary Material

Crystal structure: contains datablocks global, I. DOI: 10.1107/S1600536809014354/ci2778sup1.cif

Structure factors: contains datablocks I. DOI: 10.1107/S1600536809014354/ci2778Isup2.hkl

Additional supplementary materials:  crystallographic information; 3D view; checkCIF report

## Figures and Tables

**Table 1:** Selected bond lengths (Å)

Mn1—N2	2.234 (4)
Mn1—N4	2.237 (4)
Mn1—N3	2.244 (4)
Mn1—Cl1	2.3759 (19)
Mn1—Cl2	2.3842 (18)

**Table 2:** Hydrogen-bond geometry (Å, °)

*D*—H⋯*A*	*D*—H	H⋯*A*	*D*⋯*A*	*D*—H⋯*A*
N5—H5*A*⋯Cl2^i^	0.86	2.46	3.287 (5)	161
N1—H1⋯Cl1^ii^	0.86	2.50	3.261 (5)	147
C7—H7⋯Cl2^i^	0.93	2.78	3.681 (6)	164

Symmetry codes: (i) 
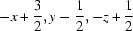
; (ii) 
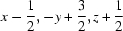
.

**Fig. 1.**
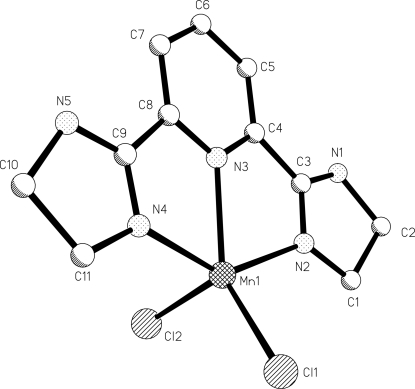
The molecular structure of the [Mn(bip)Cl2] complex, showing 50% probability displacement ellipsoids and the atom-numbering scheme. H atoms have been omitted for clarity.

**Fig. 2.**
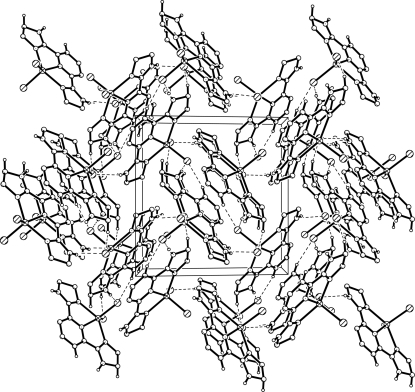
The crystal packing of [Mn(bip)Cl2] viewed along the a axis. H atoms have been omitted for clarity. Dashed lines indicate hydrogen bonds.

## supplementary crystallographic information

### Comment

The construction supramolecular architectures is currently of great interest owing to their intriguing network topologies and potential functions such as adsorption, ion exchange, shape-selective catalysis, non-linear and magnetic materials (Yaghi *et al.*, 1998; Hagrman *et al.*, 1999). The protonation and deprotonation of an imidazole ligand is believed to play an important role in the mechanism of the coordination chemistry (Bordo *et al.*, 2001). We described previously a number of such metal complexes with imidazole ligands, and concluded that hydrogen bonding involving this group influences the geometry around the metal atom and the crystallization mechanism (Ren, Ye, He *et al.*, 2004; Ren, Ye, Zhu *et al.*, 2004; Ren *et al.*,  2007; Sun *et al.*, 2008). We report here the crystal structure of the title mononuclear coordination complex, [Mn(bip)Cl_2_], where bip is 2,6-bis(4,5-dihydro-1*H*-imidazol-2-yl)pyridine.

As shown in Fig. 1, in the title compound the manganese(II) atom is five-coordinated in a distorted square-pyramidal geometry, with three N atoms from the neutral tridentate bip ligand and one Cl^-^ ion (Cl1) forming the basal plane and the other Cl^-^ ion (Cl2) in the apical position. The Mn1 atom deviates from the Cl1-N2-N3-N4 plane by 0.5633 (7) Å towards the Cl2 atom. The Mn—N bond lengths of 2.234 (4), 2.237 (4), 2.244 (4) Å are slightly shorter than those observed in metal-imidazole systems (Stupka *et al.*, 2004; Hammes *et al.*, 2005; Haga *et al.*, 1996; Böca *et al.*, 2005). The N—Mn—N bond angles lie in the range 70.69 (14)–140.86 (14)°.

Adjacent molecules are linked into a three-dimensional network by N—H···Cl and C—H···Cl hydrogen bonds (Table 1).

### Experimental

All the reagents and solvents employed were commercially available and used as received without further purification. The ligand 2,6-bis(4,5-dihydro-1*H*-imidazol-2-yl)pyridine (bip) was synthesized by literature methods (Baker *et al.*, 1991). A solution of MnCl_2_.4H_2_O (0.2 mmol, 40 mg) in acetonitrile (10 ml) was added dropwise to a stirred solution of bip (0.4 mmol, 86 mg) in methanol (10 ml) at 333 K. Yellow single crystals suitable for X-ray diffraction were obtained by slow diffusion of diethyl ether into the clear filtrate for 2 d in 60% yield. Main IR bands (KBr, cm_-1_): 3365*m*, 3251 s, 1621w, 1596*m*, 1573 s, 1523*m*, 1446 s, 1275 s, 1026*m*, 957w, 822w, 745w, 663w.

### Refinement

All the H atoms were placed in geometrically idealized positions and constrained to ride on their parent atoms, with N-H = 0.86 Å, C-H = 0.93 or 0.97 Å and *U*_iso_(H) = 1.2U_eq_(C,N).

### Crystal data

**Table:**

[MnCl_2_(C_11_H_13_N_5_)]	*F*(000) = 692
*M**_r_* = 341.10	*D*_x_ = 1.577 Mg m^−^^3^
Monoclinic, *P*2_1_/*n*	Mo *K*α radiation, λ = 0.71073 Å
Hall symbol: -P 2yn	Cell parameters from 1365 reflections
*a* = 9.297 (5) Å	θ = 2.3–23.1°
*b* = 12.686 (7) Å	µ = 1.28 mm^−^^1^
*c* = 12.383 (6) Å	*T* = 273 K
β = 100.313 (9)°	Block, yellow
*V* = 1436.9 (13) Å^3^	0.30 × 0.25 × 0.21 mm
*Z* = 4	

### Data collection

**Table:**

Bruker SMART CCD area-detector diffractometer	3317 independent reflections
Radiation source: fine-focus sealed tube	1750 reflections with *I* > 2σ(*I*)
graphite	*R*_int_ = 0.077
φ and ω scans	θ_max_ = 27.9°, θ_min_ = 2.3°
Absorption correction: multi-scan (*SADABS*, Bruker, 1998)	*h* = −10→12
*T*_min_ = 0.700, *T*_max_ = 0.774	*k* = −10→16
8507 measured reflections	*l* = −16→15

### Refinement

**Table:**

Refinement on *F*^2^	Secondary atom site location: difference Fourier map
Least-squares matrix: full	Hydrogen site location: inferred from neighbouring sites
*R*[*F*^2^ > 2σ(*F*^2^)] = 0.067	H-atom parameters constrained
*wR*(*F*^2^) = 0.187	*w* = 1/[σ^2^(*F*_o_^2^) + (0.0956*P*)^2^] where *P* = (*F*_o_^2^ + 2*F*_c_^2^)/3
*S* = 0.95	(Δ/σ)_max_ = 0.001
3317 reflections	Δρ_max_ = 0.68 e Å^−^^3^
173 parameters	Δρ_min_ = −0.53 e Å^−^^3^
0 restraints	Extinction correction: *SHELXL97* (Sheldrick, 2008), Fc^*^=kFc[1+0.001xFc^2^λ^3^/sin(2θ)]^-1/4^
Primary atom site location: structure-invariant direct methods	Extinction coefficient: 0.016 (3)

### Special details

**Table:**

Geometry. All e.s.d.'s (except the e.s.d. in the dihedral angle between two l.s. planes) are estimated using the full covariance matrix. The cell e.s.d.'s are taken into account individually in the estimation of e.s.d.'s in distances, angles and torsion angles; correlations between e.s.d.'s in cell parameters are only used when they are defined by crystal symmetry. An approximate (isotropic) treatment of cell e.s.d.'s is used for estimating e.s.d.'s involving l.s. planes.
Refinement. Refinement of *F*^2^ against ALL reflections. The weighted *R*-factor *wR* and goodness of fit *S* are based on *F*^2^, conventional *R*-factors *R* are based on *F*, with *F* set to zero for negative *F*^2^. The threshold expression of *F*^2^ > σ(*F*^2^) is used only for calculating *R*-factors(gt) *etc*. and is not relevant to the choice of reflections for refinement. *R*-factors based on *F*^2^ are statistically about twice as large as those based on *F*, and *R*- factors based on ALL data will be even larger.

### Fractional atomic coordinates and isotropic or equivalent isotropic displacement parameters (Å^2^)

**Table:**

	*x*	*y*	*z*	*U*_iso_*/*U*_eq_	
Mn1	1.09096 (8)	0.66191 (6)	0.31687 (6)	0.0431 (3)	
Cl1	1.33924 (15)	0.63750 (13)	0.29973 (12)	0.0605 (5)	
Cl2	0.96929 (17)	0.77218 (13)	0.17311 (13)	0.0697 (5)	
N1	0.9960 (5)	0.8515 (3)	0.5846 (4)	0.0519 (12)	
H1	0.9262	0.8644	0.6198	0.062*	
N2	1.1031 (5)	0.7757 (3)	0.4559 (3)	0.0492 (11)	
N3	0.9235 (4)	0.6172 (3)	0.4186 (3)	0.0360 (9)	
N4	0.9927 (4)	0.5062 (3)	0.2597 (3)	0.0458 (11)	
N5	0.8274 (5)	0.3790 (4)	0.2646 (4)	0.0542 (12)	
H5A	0.7647	0.3429	0.2923	0.065*	
C1	1.1869 (7)	0.8745 (5)	0.4878 (5)	0.0587 (16)	
H1A	1.1677	0.9266	0.4297	0.070*	
H1B	1.2911	0.8606	0.5045	0.070*	
C2	1.1298 (6)	0.9119 (5)	0.5908 (5)	0.0606 (16)	
H2A	1.1988	0.8957	0.6573	0.073*	
H2B	1.1103	0.9870	0.5881	0.073*	
C3	1.0010 (5)	0.7721 (4)	0.5141 (4)	0.0413 (12)	
C4	0.8964 (5)	0.6824 (4)	0.4970 (4)	0.0359 (11)	
C5	0.7820 (5)	0.6653 (4)	0.5540 (4)	0.0432 (12)	
H5	0.7626	0.7126	0.6069	0.052*	
C6	0.6986 (6)	0.5756 (4)	0.5292 (4)	0.0510 (14)	
H6	0.6222	0.5613	0.5662	0.061*	
C7	0.7285 (5)	0.5069 (4)	0.4493 (4)	0.0474 (13)	
H7	0.6737	0.4459	0.4325	0.057*	
C8	0.8416 (5)	0.5311 (4)	0.3951 (4)	0.0387 (11)	
C9	0.8875 (5)	0.4711 (4)	0.3049 (4)	0.0388 (11)	
C10	0.8854 (7)	0.3506 (5)	0.1671 (5)	0.0618 (17)	
H10A	0.8137	0.3615	0.1008	0.074*	
H10B	0.9186	0.2781	0.1700	0.074*	
C11	1.0136 (6)	0.4282 (5)	0.1743 (5)	0.0595 (16)	
H11A	1.1062	0.3918	0.1950	0.071*	
H11B	1.0123	0.4629	0.1043	0.071*	

### Atomic displacement parameters (Å^2^)

**Table:**

	*U*^11^	*U*^22^	*U*^33^	*U*^12^	*U*^13^	*U*^23^
Mn1	0.0457 (5)	0.0447 (5)	0.0447 (5)	−0.0006 (4)	0.0233 (4)	0.0035 (4)
Cl1	0.0490 (8)	0.0707 (10)	0.0689 (9)	0.0051 (7)	0.0298 (7)	0.0207 (8)
Cl2	0.0638 (10)	0.0713 (11)	0.0796 (11)	0.0170 (8)	0.0280 (8)	0.0339 (9)
N1	0.056 (3)	0.046 (3)	0.061 (3)	−0.016 (2)	0.029 (2)	−0.013 (2)
N2	0.050 (3)	0.049 (3)	0.054 (3)	−0.013 (2)	0.025 (2)	0.000 (2)
N3	0.041 (2)	0.034 (2)	0.035 (2)	−0.0027 (18)	0.0134 (18)	0.0013 (19)
N4	0.052 (3)	0.044 (3)	0.046 (2)	0.003 (2)	0.024 (2)	−0.002 (2)
N5	0.065 (3)	0.045 (3)	0.058 (3)	−0.009 (2)	0.025 (2)	−0.010 (2)
C1	0.062 (4)	0.048 (3)	0.071 (4)	−0.015 (3)	0.027 (3)	−0.001 (3)
C2	0.065 (4)	0.054 (4)	0.066 (4)	−0.014 (3)	0.021 (3)	−0.011 (3)
C3	0.042 (3)	0.044 (3)	0.040 (3)	−0.003 (2)	0.014 (2)	0.002 (2)
C4	0.043 (3)	0.032 (3)	0.036 (2)	−0.001 (2)	0.017 (2)	0.004 (2)
C5	0.049 (3)	0.045 (3)	0.041 (3)	−0.002 (3)	0.021 (2)	0.001 (2)
C6	0.054 (3)	0.050 (3)	0.058 (3)	−0.004 (3)	0.034 (3)	0.004 (3)
C7	0.043 (3)	0.048 (3)	0.055 (3)	−0.008 (3)	0.020 (3)	0.000 (3)
C8	0.039 (3)	0.037 (3)	0.042 (3)	0.003 (2)	0.013 (2)	0.004 (2)
C9	0.046 (3)	0.032 (3)	0.039 (3)	0.003 (2)	0.009 (2)	0.004 (2)
C10	0.076 (4)	0.056 (4)	0.056 (3)	0.012 (3)	0.017 (3)	−0.012 (3)
C11	0.066 (4)	0.064 (4)	0.054 (3)	0.003 (3)	0.027 (3)	−0.012 (3)

### Geometric parameters (Å, °)

**Table:**

Mn1—N2	2.234 (4)	C1—H1A	0.97
Mn1—N4	2.237 (4)	C1—H1B	0.97
Mn1—N3	2.244 (4)	C2—H2A	0.97
Mn1—Cl1	2.3759 (19)	C2—H2B	0.97
Mn1—Cl2	2.3842 (18)	C3—C4	1.486 (7)
N1—C3	1.340 (7)	C4—C5	1.397 (6)
N1—C2	1.450 (7)	C5—C6	1.380 (8)
N1—H1	0.86	C5—H5	0.93
N2—C3	1.291 (6)	C6—C7	1.382 (7)
N2—C1	1.489 (7)	C6—H6	0.93
N3—C4	1.333 (6)	C7—C8	1.380 (6)
N3—C8	1.333 (6)	C7—H7	0.93
N4—C9	1.289 (6)	C8—C9	1.476 (7)
N4—C11	1.487 (7)	C10—C11	1.535 (9)
N5—C9	1.352 (7)	C10—H10A	0.97
N5—C10	1.455 (7)	C10—H10B	0.97
N5—H5A	0.86	C11—H11A	0.97
C1—C2	1.542 (8)	C11—H11B	0.97
			
N2—Mn1—N4	140.86 (14)	C1—C2—H2B	111.3
N2—Mn1—N3	71.04 (14)	H2A—C2—H2B	109.2
N4—Mn1—N3	70.69 (14)	N2—C3—N1	116.8 (5)
N2—Mn1—Cl1	103.72 (12)	N2—C3—C4	118.3 (5)
N4—Mn1—Cl1	101.82 (11)	N1—C3—C4	124.8 (4)
N3—Mn1—Cl1	143.13 (12)	N3—C4—C5	122.1 (5)
N2—Mn1—Cl2	98.52 (13)	N3—C4—C3	112.1 (4)
N4—Mn1—Cl2	99.76 (12)	C5—C4—C3	125.8 (4)
N3—Mn1—Cl2	106.47 (12)	C6—C5—C4	117.7 (5)
Cl1—Mn1—Cl2	110.39 (6)	C6—C5—H5	121.2
C3—N1—C2	107.6 (4)	C4—C5—H5	121.2
C3—N1—H1	126.2	C5—C6—C7	120.1 (5)
C2—N1—H1	126.2	C5—C6—H6	119.9
C3—N2—C1	106.5 (4)	C7—C6—H6	119.9
C3—N2—Mn1	117.8 (4)	C8—C7—C6	118.6 (5)
C1—N2—Mn1	134.3 (3)	C8—C7—H7	120.7
C4—N3—C8	119.6 (4)	C6—C7—H7	120.7
C4—N3—Mn1	119.4 (3)	N3—C8—C7	121.9 (5)
C8—N3—Mn1	120.7 (3)	N3—C8—C9	110.9 (4)
C9—N4—C11	106.5 (5)	C7—C8—C9	127.1 (5)
C9—N4—Mn1	117.8 (3)	N4—C9—N5	115.8 (5)
C11—N4—Mn1	135.6 (3)	N4—C9—C8	119.6 (5)
C9—N5—C10	109.2 (5)	N5—C9—C8	124.5 (4)
C9—N5—H5A	125.4	N5—C10—C11	101.1 (5)
C10—N5—H5A	125.4	N5—C10—H10A	111.6
N2—C1—C2	103.7 (4)	C11—C10—H10A	111.6
N2—C1—H1A	111.0	N5—C10—H10B	111.6
C2—C1—H1A	111.0	C11—C10—H10B	111.6
N2—C1—H1B	111.0	H10A—C10—H10B	109.4
C2—C1—H1B	111.0	N4—C11—C10	105.6 (4)
H1A—C1—H1B	109.0	N4—C11—H11A	110.6
N1—C2—C1	102.3 (5)	C10—C11—H11A	110.6
N1—C2—H2A	111.3	N4—C11—H11B	110.6
C1—C2—H2A	111.3	C10—C11—H11B	110.6
N1—C2—H2B	111.3	H11A—C11—H11B	108.7

### Hydrogen-bond geometry (Å, °)

**Table:**

*D*—H···*A*	*D*—H	H···*A*	*D*···*A*	*D*—H···*A*
N5—H5A···Cl2^i^	0.86	2.46	3.287 (5)	161
N1—H1···Cl1^ii^	0.86	2.50	3.261 (5)	147
C7—H7···Cl2^i^	0.93	2.78	3.681 (6)	164

Symmetry codes: (i) −*x*+3/2, *y*−1/2, −*z*+1/2; (ii) *x*−1/2, −*y*+3/2, *z*+1/2.
